# Associations between erythrocyte polymorphisms and risks of uncomplicated and severe malaria in Ugandan children: A case control study

**DOI:** 10.1371/journal.pone.0203229

**Published:** 2018-09-17

**Authors:** Arthur Mpimbaza, Andrew Walakira, Grace Ndeezi, Anne Katahoire, Charles Karamagi, Samuel L. Nsobya, Stephen Tukwasibwe, Victor Asua, Philip J. Rosenthal

**Affiliations:** 1 Child Health and Development Centre, Makerere University-College of Health Sciences, Kampala, Uganda; 2 Infectious Diseases Research Collaboration, Kampala, Uganda; 3 Department of Pediatrics and Child Health, Makerere University-College of Health Sciences, Kampala, Uganda; 4 Clinical Epidemiology Unit, Department of Medicine, Makerere University-College of Health Sciences, Kampala, Uganda; 5 Department of Medicine, University of California, San Francisco, CA, United States of America; Instituto Rene Rachou, BRAZIL

## Abstract

**Background:**

Evidence for association between sickle cell and alpha thalassemia trait and severe malaria is compelling. However, for these polymorphisms associations with uncomplicated malaria, and for G6PD deficiency associations with uncomplicated and severe malaria, findings have been inconsistent. We studied samples from a three-arm case-control study with the objective of determining associations between common host erythrocyte polymorphisms and both uncomplicated and severe malaria, including different severe malaria phenotypes.

**Method:**

We assessed hemoglobin abnormalities, α-thalassemia, and G6PD deficiency by molecular methods in 325 children with severe malaria age-matched to 325 children with uncomplicated malaria and 325 healthy community controls. Conditional logistic regression was used to measure associations between specified genotypes and malaria outcomes.

**Results:**

No tested polymorphisms offered significant protection against uncomplicated malaria. α-thalassemia homozygotes (_α/_α) had increased risk of uncomplicated malaria (OR 2.40; 95%CI 1.15, 5.03, p = 0.020). HbAS and α-thalassemia heterozygous (_α/αα) genotypes protected against severe malaria compared to uncomplicated malaria (HbAS OR 0.46; 0.23, 0.95, p = 0.036; _α/αα OR 0.51; 0.24, 0.77; p = 0.001) or community (HbAS OR 0.23; 0.11, 0.50; p<0.001; _α/αα; OR 0.49; 0.32, 0.76; p = 0.002) controls. The α-thalassemia homozygous (_α/_α) genotype protected against severe malaria when compared to uncomplicated malaria controls (OR 0.34; 95%CI 0.156, 0.73, p = 0.005), but not community controls (OR 1.03; 0.46, 2.27, p = 0.935). Stratifying by the severe malaria phenotype, compared to community controls, the protective effect of HbAS was limited to children with severe anemia (OR 0.17; 95%CI 0.04, 0.65; p = 0.009) and that of _α/αα to those with altered consciousness (OR 0.24; 0.09, 0.59; p = 0.002). A negative epistatic effect was seen between HbAS and _α/αα; protection compared to uncomplicated malaria controls was not seen in individuals with both polymorphisms (OR 0.45; 0.11, 1.84; p = 0.269). G6PD deficiency was not protective against severe malaria.

**Conclusion:**

Associations were complex, with HbAS principally protective against severe anemia, _α/αα against altered consciousness, and negative epistasis between the two polymorphisms.

## Introduction

Several human genetic polymorphisms appear to protect against lethal falciparum malaria[1]. As first proposed for thalassemias by Haldane in 1949 [2], some polymorphisms that alter erythrocyte function persist in human populations as balanced polymorphisms by protecting against lethal malaria in heterozygotes, offsetting the negative consequences of the homozygous state[2]. The best characterized of these polymorphisms are common disorders of hemoglobin, with single amino acid substitutions in β-globin (hemoglobin S, C, and E) or reduced production of α- (α-thalassemia) or β- (β-thalassemia) globin [3], and deficiency in glucose-6-phosphate dehydrogenase (G6PD), which protects erythrocytes from oxidative stress[1].

Multiple studies have shown that hemoglobin S heterozygotes (HbAS) are protected against uncomplicated [4–9] and severe [4–6, 10–14] malaria due to *Plasmodium falciparum*. The mechanism of protection conferred by HbAS is incompletely understood [15, 16]. Potential mechanisms include increased splenic phagocytosis [2], premature hemolysis and parasite death[17], impaired hemoglobin digestion[18], altered cytoadherence [19], translocation of HbS-specific microRNAs [20], and growth arrest due to hemoglobin polymerization under low oxygen conditions[21]. HbC heterozygotes (HbAC) are also protected against severe malaria [12]. α–thalassemia heterozygosity (_α/αα) [12, 22–24] and homozygosity (_α/_α)[22, 23,25] are reported to protect against severe malaria; protection against uncomplicated malaria is less certain, with studies yielding contradictory results [26].

The common G6PD deficiency genotype in African populations is G6PD A- (V68M and N126D), with 8–20% of wild type enzyme activity. G6PD deficiency may protect against malaria by increasing oxidative stress in *P*. *falciparum-*infected erythrocytes, causing premature lysis [27]. A case-control study showed significant association between G6PD A- and risk of severe malaria, with protection against cerebral malaria, but increased risk of severe anemia [28]. However, compared to HbAS or α–thalassemia, associations between G6PD A- deficiency and risk of severe malaria have been less straightforward, with studies yielding inconsistent results [29, 30].

A recent case-control study of determinants of severe malaria in Uganda enabled comparison of genotypes in children with severe malaria with those in two control groups: children with uncomplicated malaria and healthy community controls [31]. The objective of this study was to determine the association between common host erythrocyte polymorphisms and malaria outcomes including uncomplicated malaria and different manifestations of severe malaria.

## Methods

### Study design

A matched-case control study was conducted to identify determinants of severe malaria in Ugandan children [31]. Cases were children, aged 6 months to less than 10 years of age with severe malaria, defined as children hospitalized at Jinja Hospital with a positive malaria blood smear (>2500 parasites/*u*l)and any of the following severe malaria criteria: 1) severe anemia (Hb<5g/dl), 2) impaired consciousness (Blantyre Coma Score <4)[32], or 3) respiratory distress (inter-costal or sub-costal recession without crackles or other evidence of pneumonia upon auscultation). Two types of controls were selected for each case. First, uncomplicated malaria controls were defined as children with fever and a blood smear positive for *P*. *falciparum* parasites (>2500 parasites/*u*l), but without clinical evidence of severe malaria, danger signs [32], or other known causes of febrile illness. Second, community controls, included to allow study of risk factors for uncomplicated malaria, were defined as children without a history of fever in the 2 weeks prior to enrollment recruited from the same village as a matched case. We sought a representative community sample, and in this high-transmission setting some community controls had asymptomatic parasitemia. Such controls were not excluded as this might have introduced bias [33, 34]. Target age groupings for matching were 6 to <12 months, 1 to <3 years, 3 to <5 years, and ≥5 years.

### Malaria microscopy

Thick blood smears were stained with 10% Giemsa for 10 minutes and were initially read as positive or negative for malaria parasites by health facility staff as part of their routine practice. Enrollment of severe malaria cases and uncomplicated malaria controls was confirmed upon ascertainment of a positive malaria smear and adequacy of parasite density by a study laboratory technician. Thick blood smears reported as positive were sent to a central laboratory to be read by two expert microscopists for determination of parasite densities. Results were considered final if the first and second expert readings agreed on parasites densities (< 25% level of disagreement). If the first and second expert readings were discordant, a third expert, reader broke the tie.

### Characterization of human genotypes

For each child, a blood sample was collected into an EDTA tube, and DNA was purified as previously described [35]. Characterization of genotypes for hemoglobin (HbS and HbC), the α-thalassemia 3.7 kb deletion, and the common African G6PD deficiency genotype (G6PD A-)was performed with PCR-based assays, as described previously[35]. Outcomes for each tested genotype were categorized as wild type, heterozygous, or homozygous. For α-thalassemia, the αα/αα genotype represented wild type, _α/αα the heterozygote (silent carrier), and _α/_α the homozygote (trait). For G6PD A-, females were considered wild type (A/A), heterozygous (A/A-) or homozygous (A-/A-), whereas males were considered wild type (A) or hemizygous (A-).

### Sample size estimation

Sample size was powered to detect a difference in the odds of α-thalassemia heterozygosity (_α/αα) between severe malaria cases and either uncomplicated malaria or healthy community controls. The population prevalence of _α/αα was estimated at 44%, and we projected exclusion of 5% of enrolled patients due to false positive malaria tests. Based on these assumptions, we required a sample size of 325 children in each arm to detect a minimum odds ratio of 0.5 for risk of severe malaria among children with α-thalassemia heterozygosity, assuming power of 80%, and alpha of 0.05.

### Analysis

Data were analyzed using STATA (version 14; STATA Corp.). Age of participants was summarized using medians. Gender, ethnicity (Nilotic vs. Bantu, based on the mother’s tribe as reported by the caregiver), and different manifestations of severe malaria were reported as proportions. Differences in distribution of baseline characteristics between cases and controls were compared using the Wilcoxon signed-rank test and the chi-square test for continuous and categorical data, respectively. Prevalence of genotypes and allele frequencies were calculated for different groups. Adherence to Hardy–Weinberg Equilibrium (HWE) was assessed in community controls based on observed versus expected genotypes; an exact test (p<0.05) was considered a violation of HWE (S1 Table). Conditional logistic regression with adjustment for age (to account for residual confounding) and caretaker’s ethnicity (Nilotic vs. Bantu), a potential confounder [35], was used to measure associations between each polymorphism and risks of uncomplicated malaria (compared to community controls) and severe malaria. For association with severe malaria (including the sub-groups of severe anemia and impaired consciousness), comparisons were with uncomplicated malaria and community control groups. To assess if _α/αα interacted with HbAS, we used two different approaches. First, we presented the individual effects of each polymorphism and the joint effects relative to wild type as the reference category in a conditional logistic regression model, determining the relative excess risk due to interaction, attributable proportion, and synergy index on an additive scale. As these measures were developed for risk rather than preventive factors[36], we recoded outcomes such that severe malaria was the reference and uncomplicated malaria associated with increased risk, resulting in transformation of the exposure variables as risk and not protective factors. Second, the significance of the product terms between HbAS and _α/αα in the regression model was assessed using the Wald statistic.

### Ethics approval

Institutional approval was obtained from the Uganda National Council of Science and Technology and the Institutional Review Boards of the College of Health Sciences, Makerere University, and the University of California San Francisco. Written informed consent was obtained from each caregiver. Interviews were conducted in accordance with good clinical practice, applicable patient privacy requirements, and the guiding principles of the Declaration of Helsinki.

## Results

### Study participants

**Fig 1:**
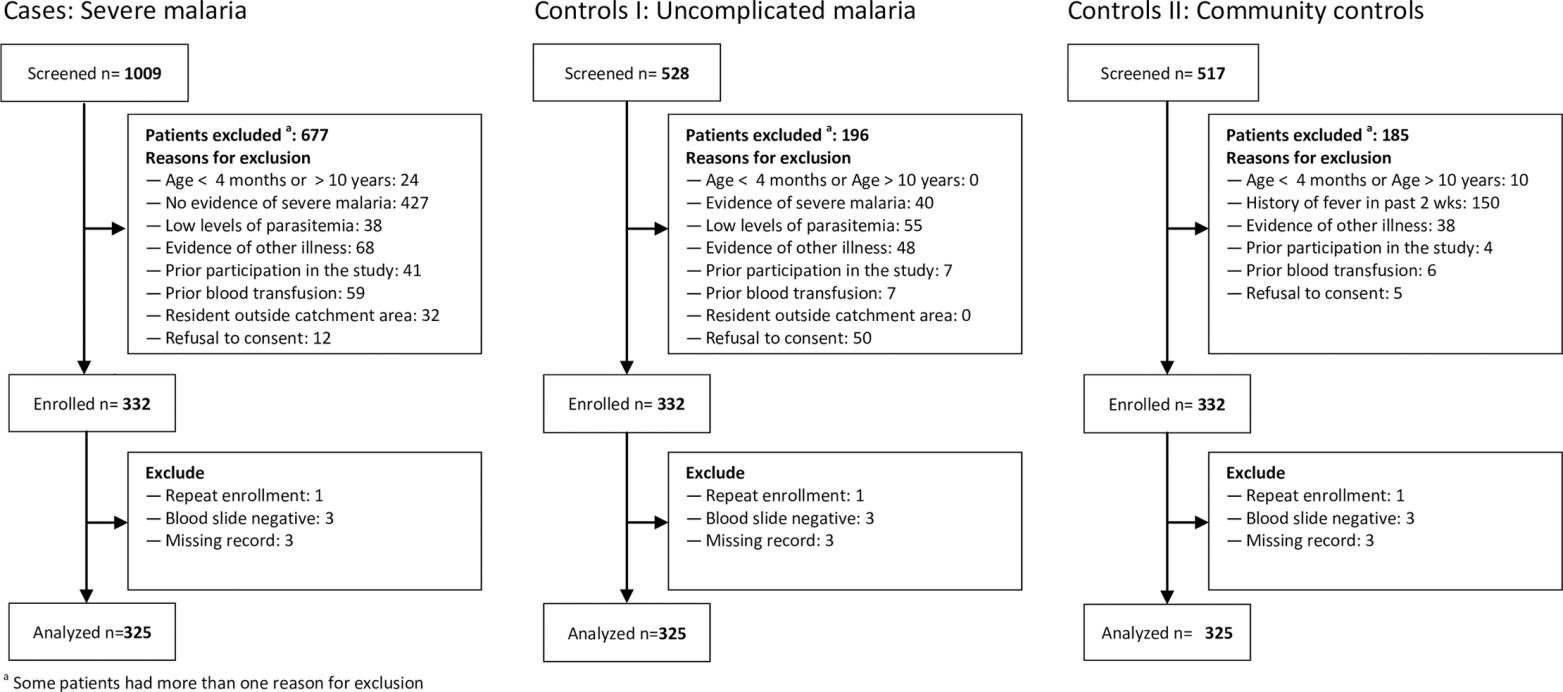
Study profile.

A total of 2054 children were screened, including 1009 severe malaria cases, 528 with uncomplicated malaria, and 517 community controls (Fig 1). Among cases, the most common reason for exclusion was lack of specified severe malaria criteria (427, 67.0%).We excluded 196 (37%) uncomplicated malaria controls; low parasitemia, evidence of another illness, and evidence of severe malaria were the most common reasons for exclusion. We excluded 185 (35.7%) community controls; history of fever in the past two weeks was the most common reason for exclusion. Of 332 sets of cases and controls initially enrolled, 7 sets were excluded; 3 had a case with a negative confirmatory malaria test, 3 a missing record, and 1 a severe malaria case accidentally enrolled twice, but considered for analysis only once, leaving 325 children in each group eligible for analysis.

### Characteristics of the study groups

The median ages for children in the 3 study groups were similar, but severe malaria cases were younger than both community (median 1.98 vs. 2.15 years, p<0.001)and uncomplicated malaria (2.13 years, p< 0.001) controls (Table 1).The distribution of age groups based on matching criteria among cases and controls was similar, but the severe malaria case group had a larger proportion of children less than one year of age(20.0%) compared to community (13.4%) and uncomplicated malaria (14.1%) controls. Distributions of females and Nilotics were similar across cases and controls (Table 1). Matching by age group was successful for 73.4% of severe malaria and uncomplicated malaria, 72.7% of severe malaria and community, and 71.0% of uncomplicated malaria and community case-control pairs.

**Table 1:** Characteristics of the study population.

Variable		Community controls N = 325	Uncomplicated malaria controls N = 325	Severe malaria cases N = 325	p-value^a^	p-value^b^	p-value^c^
Age in years (IQR)		2.15 (1.35–3.17)	2.13 (1.34–3.15)	1.98 (1.11–2.95)	0.449	<0.001	<0.001
Age category	< 1year	44 (13.5%)	46 (14.1%)	65 (20.0%)	0.926	<0.001	<0.001
	1 to < 3 years	183 (56.3%)	184 (56.6%)	181 (55.6%)			
	3 to < 5 years	78 (24.0%)	70 (21.5%)	57 (17.5%)			
	5 years or more	20 (6.1%)	25 (7.6%)	22 (6.7%)			
Females, n (%)		174 (53.7%)	157 (48.4%)	152 (46.7%)	0.172	0.07	0.624
Ethnicity (Nilotic versus Bantu)		15 (4.6%)	17 (5.2%)	11 (3.4%)	0.723	0.393	0.200

^a^ Community controls vs. uncomplicated malaria controls

^b^ community controls vs. severe malaria

^c^ uncomplicated malaria controls vs. severe malaria.

### Characteristics of children with severe malaria (cases)

Among the 325 children with severe malaria, 446 severe malaria criteria were observed, most commonly severe anemia (Hb<5g/dl; 49.1%), impaired consciousness (32.3%), and respiratory distress (18.6%). Of the 325 children with severe malaria, 222 (68.3%) of them presented with a single severe malaria-defining syndrome, including severe anemia (64.9%), impaired consciousness (26.6%) and respiratory distress (8.5%). In the 103 children presenting with more than one defining criterion, severe anemia with unconsciousness was the most common (37.9%), followed by respiratory distress with unconsciousness (24.3%).

### Prevalence of erythrocyte polymorphisms

Mutant genotypes were common for all polymorphisms studied, except HbC, with many more heterozygous than homozygous alleles identified (Table 2).Among community controls, allele frequencies were 6.2% for HbS, 0% for HbC, 22.3% for α-thalassemia, and 10.2% for G6PDA; none of the allele distributions violated Hardy Weinberg equilibrium (S1 Table).

**Table 2:** Genotype and allele frequencies for specified gene polymorphism among cases and controls.

Variable	Genotype	Community controls N = 325			Uncomplicated malaria controls N = 325			Severe malaria cases N = 325		
		Number tested	Prevalence n (%)	Allele frequency	Number tested	Prevalence n (%)	Allele frequency	Number tested	Prevalence n (%)	Allele frequency
HbAS	AA	317 (97.5%)	275 (86.7%)	6.6%	314(96.6%)	282 (89.8%)	5.8%	314 (96.6%)	297 (94.5%)	3.1%
	AS		42 (13.2%)			27 (8.6%)			14 (4.4%)	
	SS		0			5 (1.5%)			3 (0.9%)	
HbAC	AA	320 (97.5%)	0	0	316 (96.3%)	0	0	317 (96.6%)	0	0
	AC		0			0			0	
	CC		0			0			0	
α-thalassemia	αα/αα	304 (93.5%)	184 (60.5%)	22.3%	300 (92.3%)	167 (55.6%)	27.3%	302 (92.9%)	223 (73.8%)	15.6%
	_ α/αα		104 (34.2%)			102 (34.0%)			64 (21.1%)	
	_α/_α		16 (5.2%)			31 (10.3%)			15 (4.9%)	
G6PD-females	Wild type	172 (98.8%)	147 (85.4%)	8.7%	151 (96.1%)	118 (78.1%)	13.2%	148 (97.3%)	113 (76.3%)	13.5%
	Heterozygous		20 (11.6%)			26 (17.2%)			30 (20.2%)	
	Homozygous		5 (2.9%)			7 (4.6%)			5 (3.3%)	
G6PD-males	Wild type	142 (94.6%)	122 (85.9%)	7.5%	162 (97.0%)	132 (81.4%)	18.5%	166 (95.9%)	140 (84.3%)	15.6%
	Hemizygous		20 (14.0%)			30 (18.5%)			26 (15.6%)	

### Association between host polymorphisms and risks of uncomplicated malaria compared to community controls

Hemoglobin S heterozygotes had marginal protection against uncomplicated malaria (OR 0.60; 95%CI 0.34, 1.03, p = 0.065) (Table 3). α-thalassemia heterozygotes (_α/αα) did not demonstrate a significant association with uncomplicated malaria (OR 1.11; 95% CI 0.74, 1.56, p = 0.697), and α-thalassemia homozygotes (_α/_α) had increased risk of uncomplicated malaria (OR 2.40; 95% CI 1.15, 5.03, p = 0.020). G6PD A- was not associated with risk of uncomplicated malaria in female heterozygotes (OR 1.43; 95% CI 0.65, 3.14, p = 0.370), female homozygotes (OR 3.21; 95% CI 0.28, 35.7, p = 0.341), or male hemizygotes (OR 1.52; 95% CI 0.67, 3.41, p = 0.307).

**Table 3:** Association between specific host polymorphisms and risk of malaria infection and severe malaria.

Host polymorphism	Prevalence n/N (%)			Adjusted analysis		
	Community controls	Uncomplicated malaria	Severe malaria cases	Uncomplicated malaria cases Vs. Community controls	Severe malaria cases Vs. Uncomplicated malaria controls	Severe malaria cases Vs. Community Controls
				OR (95%CI); p-value^a^	OR (95%CI); p-value^b^	OR (95%CI); p-value^c^
**All children**						
AS	42/317 (13.2%)	27/309 (8.7%)	14/311 (4.5%)	0.60 (0.34, 1.03); 0.065	0.46 (0.23, 0.95); 0.036	0.23 (0.11, 0.50); <0.001
_α/αα	104/304 (34.2%)	102/300 (34.0%)	64/302 (21.1%)	1.11 (0.74, 1.56); 0.697	0.51 (0.24, 0.77); 0.001	0.49 (0.32, 0.76); 0.002
_α/_α	16/304 (5.2%)	31/300 (10.3%)	15/302 (4.9%)	2.40 (1.15, 5.03); 0.020	0.34 (0.16, 0.73); 0.005	1.03 (0.46, 2.27);0.935
G6PD-Females Heterozygous A/A-	20/172 (11.6%)	26/151 (17.2%)	30/148 (20.2%)	1.43 (0.65, 3.14); 0.370	1.98 (0.78, 5.03); 0.150	1.94 (0.75, 4.99); 0.169
G6PD-Females Homozygous A-/A-	5/172 (2.9%)	7/151 (4.6%)	5/148 (3.3%)	3.21 (0.28, 35.7); 0.341	0.42 (0.07, 2.64); 0.350	1.16 (0.25, 5.40); 0.847
G6PD Males Hemizygote A-	20/142 (14.0%)	30/162 (18.5%)	26/166 (15.6%)	1.52 (0.67, 3.41); 0.307	0.99 (0.40, 2.42); 0.986	0.86 (0.35, 2.08);0.745
**Children with severe malaria attributed to severe anemia only**^d^						
AS	18/135 (13.3%)	11/132 (8.3%)	6/130 (4.6%)	NA	0.47 (0.13, 1.65); 0.245	0.17 (0.04, 0.65); 0.009
_α/αα	36/129 (27.9%)	45/124 (36.2%)	33/128 (25.7%)	NA	0.66 (0.35, 1.23); 0.193	0.78 (0.38, 1.57); 0.487
_α/_α	6/129 (4.6%)	13/124 (10.4%)	10/128 (7.8%)	NA	0.55(0.21, 1.44); 0.226	1.60 (0.51, 5.03); 0.415
G6PD-Females Heterozygous A/A-	7/72 (9.7%)	12/64 (18.7%)	11/67 (16.4%)	NA	1.91 (0.45, 7.99); 0.372	0.83 (0.15, 4.40); 0.835
G6PD-Females Homozygous A-/A-	3/72 (4.1%)	5/64 (7.8%)	2/67 (2.9%)	NA	0.22 (0.02, 2.39); 0.218	0.29 (0.001, 96.1); 0.678
G6PD Males Hemizygote A-	9/61 (14.7%)	10/68 (14.7%)	10/66 (15.1%)	NA	3.24 (0.56, 21.2); 0.179	0.25 (0.02, 2.42); 0.233
**Children with severe malaria attributed to impaired consciousness only**^d^						
AS	7/84 (8.3%)	4/83 (4.8%)	6/84 (7.1%)	NA	1.34 (0.37, 4.83); 0.647	0.80 (0.24, 2.66); 0.724
_α/αα	34/80 (42.5%)	26/80 (32.5%)	13/79 (16.4%)	NA	0.31 (0.12, 0.80); 0.016	0.24 (0.09, 0.59); 0.002
_α/_α	7/80 (8.7%)	10/80 (12.5%)	2/79 (2.5%)	NA	0.08 (0.009, 0.72); 0.025	0.47 (0.08, 2.63); 0.396
G6PD-Females Heterozygous A/A-	2/44 (4.5%)	7/39 (17.9%)	6/35 (17.1%)	NA	^e^	^e^
G6PD-Females Homozygous A-/A-	1/44 (2.2%)	1/39 (2.5%)	0	NA	^e^	^e^
G6PD Males Hemizygote A-	5/40 (12.5%)	11/45 (24.4%)	9/48 (18.7%)	NA	0.37 (0.05, 2.41); 0.305	1.21 (0.29, 5.02); 0.784

NA: Not applicable

^a^ Adjusted analysis: uncomplicated malaria cases vs. community controls

^b^ Adjusted analysis: severe malaria cases vs. uncomplicated malaria controls

^c^Adjusted analysis: severe malaria cases vs. community controls

^d^ Analysis limited to case control pairs; cases are defined by the listed severe malaria phenotype

^e^Insufficient numbers resulting in unintelligible effect estimates and confidence intervals

### Association between host polymorphisms and risks of severe malaria compared to uncomplicated malaria controls

Hemoglobin S heterozygous (OR 0.46; 95% CI 0.23, 0.95, p = 0.036), _α/αα (OR 0.51; 95% CI 0.24, 0.77, p = 0.001) and _α/_α(OR 0.34; 95% CI 0.16, 0.73, p = 0.005) genotypes demonstrated protection against severe malaria when compared to uncomplicated malaria controls (Table 3). Upon sub-group analysis by type of severe malaria, the protective effect of HbAS was not demonstrated for severe anemia or impaired consciousness. In contrast, the protective effects of _α/αα and _α/_α were significant for children with impaired consciousness, a surrogate for cerebral malaria (OR 0.31; 95% CI 0.12, 0.80, p = 0.016 and OR 0.08; 95% CI 0.009, 0.72, p = 0.025, respectively), but not for children with severe anemia (OR 0.66; 95% CI 0.35, 1.23, p = 0.193 and OR 0.55; 95% CI 0.21, 1.44, p = 0.226, respectively). G6PD A- was not associated with protection against severe malaria or against specific types of severe malaria in both females and males.

### Association between host polymorphisms and risks of severe malaria compared to community controls

Hemoglobin S heterozygotes demonstrated protection against severe malaria when compared to community controls (OR 0.23; 95% CI 0.11, 0.50, p <0.001; Table 3). Similarly, _α/αα demonstrated protection against severe malaria (OR 0.49; 95% CI 0.32, 0.76, p = 0.002), whereas _α/_α did not demonstrate this protection (OR 1.04; 95% CI 0.45, 2.41, p = 0.918). Upon sub-group analysis by severe malaria phenotype, when compared to community controls, HbAS protection was limited to children with severe anemia (OR 0.17; 95% CI 0.04, 0.65, p = 0.009). In contrast, the protective effect of _α/αα was limited to children with impaired consciousness (OR 0.24; 95% CI 0.09, 0.59, p = 0.002). The _α/_α genotype was not protective against any of the severe malaria phenotypes. G6PD A- was not associated with protection against severe malaria or against specific types of severe malaria in both females and males.

### Interaction between HbAS and _α/αα

Based on the regression model, compared to wild type individuals, HbAS (OR 0.28; 95%CI 0.10, 0.76, p = 0.013) and _α/αα (OR 0.45; 95% CI 0.29, 0.70, p<0.001) demonstrated significant protection against severe malaria, whereas the co-inheritance of both HbAS and _α/αα did not (OR 0.45; 95% CI 0.11, 1.84, p = 0.269), all compared to uncomplicated malaria controls (Table 4). Upon recoding the outcome such that the exposure variables became risk and not protective factors, the three measures of additive interaction, relative excess risk due to interaction (-2.07; 95% CI -6.40, 2.24), attributable proportion (-0.84; 95% CI -34.2, 1.73) and synergy index (0.41; 95% CI -34.4, 1.73) all indicated significant negative interaction on the additive scale. Inclusion of the product of HbAS and _α/αα in the regression model was not statistically significant (Wald statistic 0.175), possibly due to small sample size. Overall, negative epistasis was observed, whereby combined HbAS and _α/αα genotype modified the protective effects of each individual polymorphism.

**Table 4:** Association between α-thalassemia heterozygote (_α/αα) and Hemoglobin AS including interaction terms.

Variables	Odds Ratio (95%CI)	p-value
**Model A. Analysis of individual and joint categories of HbAS and _α/αα_. Outcome: severe (1) vs. uncomplicated (0) malaria**		
Normal (HbAA and αα/αα)	**Ref**	**1**
HbAA and _α/αα	0.45 (0.29, 0.70)	<0.001
HbAS and αα/αα	0.28 (0.10, 0.76)	0.013
HbAS and _α/αα	0.45 (0.11, 1.84)	0.269
**Model B. Analysis of individual and joint categories of HbAS and _α/αα_. Outcome: uncomplicated (1) vs. severe malaria (0)**		
Normal (HbAA and αα/αα)	Ref	1
HbAA and _α/αα	2.18 (1.41, 3.38)	<0.001
HbAS and αα/αα	3.47 (1.30, 9.27)	0.013
HbAS and _α/αα	2.19 (0.54, 8.89)	0.269
**Model C. Including an interaction term between HbAS and αα/α_. Outcome: severe (1) vs. uncomplicated (0) malaria**		
Hemoglobin S heterozygote (HbAS)	0.28 (0.10, 0.76)	0.013
α-thalassemia heterozygote (_α/αα)	0.45 (0.29, 0.70)	<0.001
HbAS#_α/αα ^a^	3.45 (0.57, 20.7)	0.175

Measures of interaction on an additive scale (based on Model B)

Relative excess risk due to interaction: -2.07 (95% CI, -6.40, 2.24)

Attributable proportion due to interaction: -0.84 (95% CI -3.42, 1.73)

Synergy index: 0.41 (95% CI 0.04, 3.88)

^a^ Interaction term of HbAS and _α/αα

#: Operator symbol for creating an interaction between two variables.

## Discussion

Polymorphisms that alter the sequence of hemoglobin, decrease hemoglobin production, or decrease the activity of G6PD have been associated with changes in malaria risk, but results from a range of studies have varied. We evaluated the impacts of common erythrocyte polymorphisms on malaria risk, taking advantage of a trial that included children with clinically well-characterized severe malaria and two sets of matched controls, children with uncomplicated malaria and healthy community members. Overall, HbAS, _α/αα, and _α/_α were protective against severe malaria, but protection against different severe malaria phenotypes varied. Specifically, HbAS was protective against severe malarial anemia whereas _α/αα and _α/_α protected against impaired consciousness. In addition, a negative epistatic effect was seen between HbAS and _α/αα such that individuals with both polymorphisms were not protected against severe malaria.

HbAS demonstrated a trend toward protection against uncomplicated malaria compared to community controls, a finding consistent with previous case control [4] and prospective [5–9, 37] studies. _α/αα was not protective against uncomplicated malaria, consistent with most studies [5, 8, 22, 38–40], but differing from one study in Tanzania [41]. In contrast, _α/_α was associated with increased risk of uncomplicated malaria, as demonstrated previously in Vanuatu[39] and Tanzania [42]. As in those prior studies, the association between _α/_α and increased risk of uncomplicated malaria was more pronounced in younger children (OR 3.33; 95% CI 0.85, 13.0, p = 0.083 for children under 4 years of age; OR 1.85; 95% CI 0.16, 21.2, p = 0.618 for those over 4 years of age). These findings suggest an age dependent effect of immunity contributing to the interaction of _α/_α with risk of severe malaria [41]. A similar age dependent effect on HbAS protection against uncomplicated malaria has been demonstrated, with protection more pronounced in older children [37, 43].

HbAS demonstrated protection against severe malaria when compared to both uncomplicated malaria and community controls. These findings are consistent with prior case controls studies, in which HbAS demonstrated protection against severe malaria when compared to uncomplicated malaria [37, 44] and healthy community [10–12, 44] controls. Two cohort studies in Kenya also demonstrated that HbAS protected against severe malaria when incidence was compared to that in healthy controls [5, 6]. α-thalassemia heterozygotes (_α/αα) were also protected against severe malaria when compared to both uncomplicated and community controls, consistent with findings from previous case control[12, 23, 24] and cohort [5] studies. α-thalassemia homozygotes (_α/_α) were protected against severe malaria, but only when compared to uncomplicated malaria controls, unlike other studies that also demonstrated protection compared to community controls [12, 23]. The lack of association between _α/_α and severe malaria when compared to community but not uncomplicated malaria controls might be explained by different selection biases for the different control groups, with community controls biased toward the null.

Upon stratification by severe malaria phenotype, the protective effect of HbAS was limited to cases with severe anemia. This association might be attributed to the HbAS phenotype limiting multiplication of parasites in erythrocytes, thereby limiting erythrocyte destruction and progression to severe anemia [45]. These results contrast with results from a cohort study conducted in Kenya[6], and two case control studies, one in Ghana and the other a multicenter centers African study, which demonstrated HbAS protection against both severe malarial anemia and cerebral malaria[12, 44]. Differences may be explained by larger sample sizes for the other studies. For _α/αα, the protective effect was limited to children with impaired consciousness. This association may be attributed to mechanisms of malaria protection by α-thalassemia, including abnormal expression of Pfemp1, which mediates adherence of parasitized erythrocytes to brain endothelial cells[46]. However, other studies from East and West Africa [12, 22] showed that protection with _α/αα was limited to patients with severe anemia. Lack of _α/αα protection against severe anemia in our study might be explained by _α/αα protection against rapid, but not slowly progressive anemia [12], with slow progression more prevalent in our study setting characterized by high transmission.

Among children with HbAS, co-inheritance of _α/αα was more common (26.1%) among community controls; the same prevalence was observed in healthy blood donors in Cameroon [47]. Importantly, a negative epistatic effect was seen between HbAS and _α/αα for protection against severe malaria. This negative interaction between HbAS and _α/αα has been described previously [48] and is potentially explained by reversal of some protective changes attributed to HbAS in the setting of α-thalassemia. Additionally, α-thalassemia has been associated with reduced intraerythrocytic concentrations of HbS, potentially countering protective effects of HbAS against malaria[49]. Studies of associations between the G6PD A- genotypeand severe malaria have been less consistent than for the other studied polymorphisms, with reports of protective effects in females [50], in males[51], in both[52], or no protection[53]. Reasons for discrepancies are uncertain, but the lack of significant findings in our study may have been explained by a relatively small sample size.

Our study findings add to the literature demonstrating that HbAS, _α/αα and _α/_α protect against severe malaria. However, the effects of these polymorphisms varied depending on the type of severe malaria. Co-inheritance of HbS and _α/αα had a negative epistatic effect for protection against severe malaria. Longitudinal studies with larger sample sizes should be helpful in better characterizing the complex and differing impacts of these polymorphisms. Consideration of additional human polymorphisms of interest including those regulating expression of endothelial receptors such as CD36 and endothelial protein C receptor [54], and those regulating the inflammatory response, including complement receptor 1, nitric oxide synthase 2, and tumor necrosis factor-α[55] will enable a fuller understanding of the impact of human genetics on risks of uncomplicated and severe malaria.

## Supporting information

S1 Table Adherence to Hardy-Weinberg equilibrium among community controls. (TIF)

S1 Dataset Severe malaria case control study dataset. (DTA)
