# Supplementary figures and images for: Associations between erythrocyte polymorphisms and risks of uncomplicated and severe malaria in Ugandan children: A case control study
Source: PLoS One. 2018 Sep 17;13(9):e0203229. doi: 10.1371/journal.pone.0203229 (PMC6141089; doi:10.1371/journal.pone.0203229)

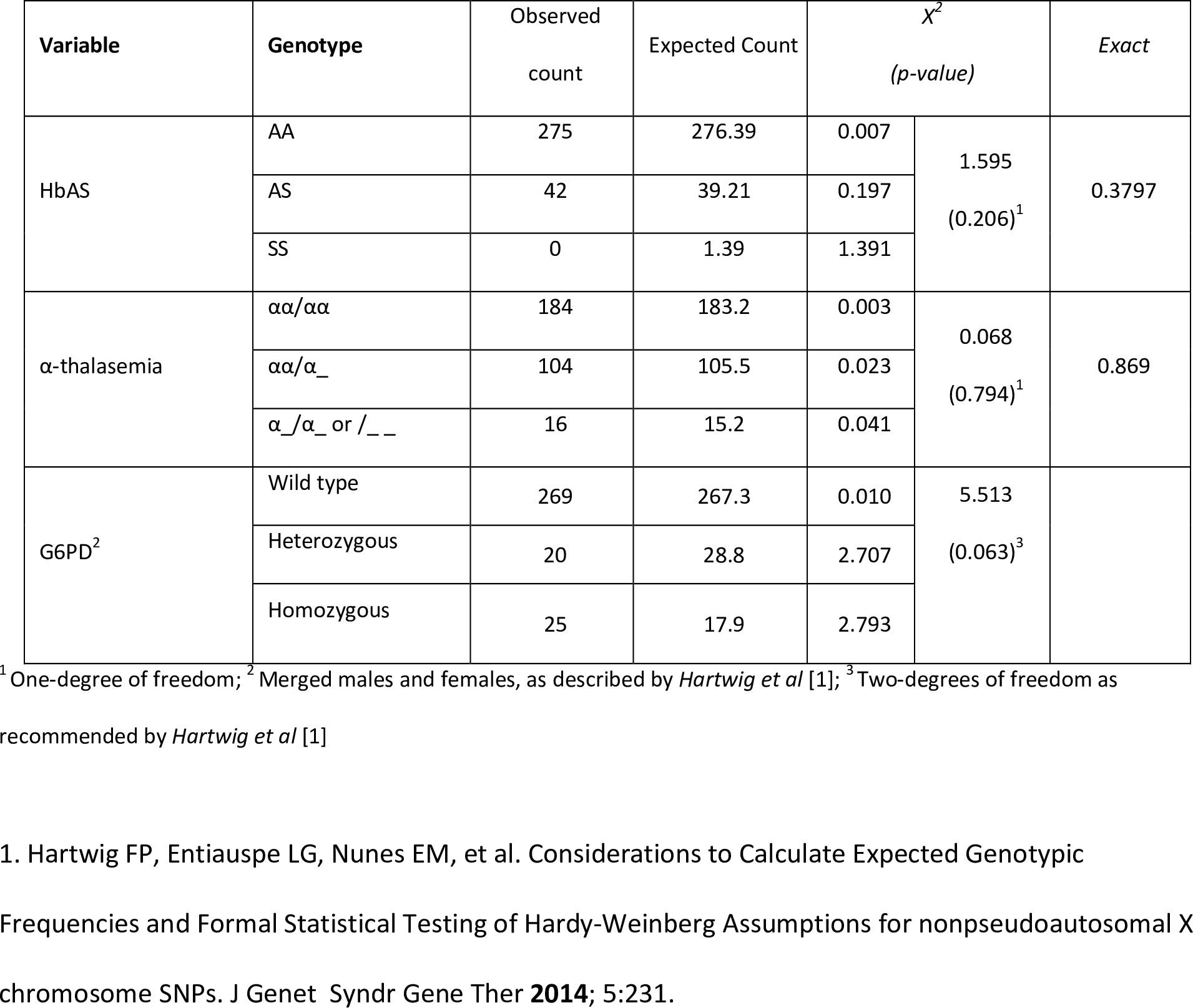

Supplement: S1 Table — (TIF) [file pone.0203229.s001.tif]
